# Victimization among children and adolescents accessing the Meyer pediatric hospital: A retrospective study

**DOI:** 10.1111/jcap.12337

**Published:** 2021-06-10

**Authors:** Annalaura Nocentini, Giada Fiorentini, Francesca Maffei, Rosanna Martin, Stefania Losi, Caterina Teodori, Tiziana Pisano, Sara Gori, Lisa De Luca, Ersilia Menesini

**Affiliations:** ^1^ Department of Education, Languages, Intercultures, Literatures and Psychology University of Florence Florence Italy; ^2^ Child Psychology Unit A. Meyer Children's Hospital Florence Italy; ^3^ Pediatric Gynecology Unit, Group for Prevention and Treatment of Abuse of Children and Adolescents (GAIA) A. Meyer Children's Hospital Florence Italy; ^4^ Child Psychology Unit, Group for Prevention and Treatment of Abuse of Children and Adolescents (GAIA) A. Meyer Children's Hospital Florence Italy; ^5^ Child and Adolescent Psychiatry Unit A. Meyer Children's Hospital Florence Italy

**Keywords:** children and adolescents, emergency departments, peer victimization

## Abstract

**Problem:**

The consistent prevalence and occasionally severe consequences of bullying and victimization suggest the need to include a more accurate assessment of these episodes within the Emergency Departments (ED). However, the literature on mental health related symptoms of bullying/victimization treated in the ED is still scarce. The aim of this study is to assess the prevalence of peer victimization amongst children and adolescents referred to an Italian Pediatric Emergency Department. Differences between Hospital Departments, type of victimization and ages are tested.

**Methods:**

A retrospective observational study was conducted with 705 subjects. The age range was from 6 to 18 years old (*M* = 13.09; *SD* = 3.048).

**Findings:**

15.3% of the sample reported to be victimized (8.2% occasionally; 7.1% systematically). For the Child and Adolescent Psychiatry Unit, we found a significant association between peer victimization and being adolescent (Fisher's *p* = 0.003). In addition, a significant association was found between verbal victimization and Child and Adolescent Psychiatry Unit (Fisher's *p* = 0.02) and physical victimization and Child Abuse Department (Fisher's *p* < 0.001).

**Conclusion:**

Findings suggest the importance of an accurate assessment of victimization experiences of children and adolescents with access to ED, to prevent future re‐victimization and crystallization of symptoms across time.

## 1 INTRODUCTION

Bullying and victimization constitute universal public health concerns that affect a significant proportion of children and adolescents around the world, having severe consequences for physical and psychological health (Menesini & Salmivalli, 2017; Wolke & Lereya, 2015; Zarate‐Garza et al., 2017). The growing awareness of the prevalence and consequences of bullying and victimization in the general population has led to consider the possibility to include a more accurate assessment of these episodes within the Emergency Departments (ED) (Waseem et al., 2017). This can have several consequences and benefits in terms of taking charge of the victims with severe consequences, prevent possible re‐victimization episodes, define more individualized interventions carried out by the ED or by the community services, help school in defining protocols for secondary and tertiary prevention. However, the literature on health and mental health related symptoms of bullying/victimization that are treated in the ED is still scarce, particularly in Italy. The current study aims to reduce this gap investigating the nature of victimization episodes reported by the children and adolescents who accessed to three different EDs of the Meyer Pediatric Hospital: a Psychological Department, a Child and Adolescent Psychiatry and Neurorehabilitation, and a Child and Adolescent Abuse Group Unit.

### 1.1 Physical health and mental health consequences of bullying and victimization

The physical health consequences of bullying can be immediate, such as physical injury, or they can involve long‐term effects, such as headaches, sleep disturbances, somatization and psychological consequences (National Academies of Sciences Engineering and Medicine, 2016). The study of physical injury as direct consequences of physical bullying, including hitting and kicking or tripping and pushing, received a very limited attention (Johnson et al., 2020) as compared to the attention paid to somatic and psychological consequences. Psychosomatic problems and a general poor physical health with a variety of symptoms, such as headache, backache, abdominal pain, skin problems, sleeping problems, bedwetting, or dizziness have been found to be associated with being victimized, in particular for boys (Gini & Pozzoli, 2013). A very consistent literature focused on psychological consequences related to being victimized, including depression, anxiety, self‐harm and non‐suicidal self‐injury, and suicidal ideation and attempts (Moore et al., 2017). Few studies analyzed the role that the type of victimization has on health consequences. Some of them reported no significant differences between direct and indirect bullying on psychological (Yen et al., 2013, 2014) and somatic complaints (Baldry, 2004). As regard cyberbullying, a meta‐analysis has suggested that it is strongly related to psychological consequences such as depression and risks of suicidality (Yen et al., 2014; Zhou et al., 2013).

The majority of the studies on consequences of victimization used community samples and longitudinal prospective designs. However, little research has investigated health and mental health related symptoms of bullying/victimization that are treated in the ED (Waseem et al., 2017). This literature distinguished studies focused on physical injuries or behavioral issues, and psychiatric and psychological symptoms related to bullying experiences treated in the ED.

As pertain to the first group all the studies were conducted in US samples. In particular, in a sample of urban adolescents referred to ED for physical injuries, 40% attributed the injury due to a bullying situation (Johnson et al., 2020). Amanullah et al. (2014) found that 12.5% of intentional injuries treated in the ED occurred in schools and that younger adolescents were more likely to attribute their injury to bullying situations. One study suggested that, among 591 youth treated in the ED due to behavioral issues, 24% reported bullying victimization (Waseem et al., 2013).

As pertain the second group, several studies showed evidence that a large proportion of children and adolescents who have experienced bullying come into contact with mental health services, although with a high rate of variation between studies. Kumpulainen et al. (2001) found that 44% of “bully/victims,” 42% of “bullies,” and 24% of “victims” had had contact with mental health professionals compared with 13% of controls. In a sample of 52 adolescents from a psychiatric outpatient service in England Salmon et al. (2000) reported that 27% of participants had been bullied. Bullying resulted a common problem in Canadian children and adolescents presenting to the ED with a mental health complaint, as approximately 77% reported experiencing bullying during their lifetimes (Alavi et al., 2015). Another study conducted in Canada reported that the prevalence of bullying was 26.9% among adolescents referred by the ED for urgent psychiatric assessment (Roberts et al., 2016). In a clinical sample of 508 adolescents admitted to a Hospital Department of Psychiatry in Finland, 29.3% of boys and 38.3% of females were victims of bullying (Luukkonen et al., 2010). In a clinical psychiatric sample in Norway, 19% reported being bullied often or very often, and 51% reported being bullied from time to time (Hansen et al., 2014). In Italy, studies have investigated the effect of victimization on children and adolescent mental health in community samples (Gini, 2008; Menesini & Nocentini, 2015), but few studies yet were conducted on clinical populations at the EDs (Pisano et al., 2020). With the increasing number of children and adolescents being admitted to EDs with health complaints (Dolan et al., 2011; Pittsenbarger & Mannix, 2014), professionals should be more aware that these complaints could be also related to potential bullying situations. The ED represents an important location for secondary and tertiary prevention, intervening once an individual has been involved in violence. Having more information about the nature of the victimization cases entering into the EDs might be helpful to better define protocols for the primary and secondary prevention in schools. Besides, the ED may have a role to prevent the re‐victimization through the guidance from ED providers and to facilitate the access to hospital or community interventions able to monitor the wellbeing of the victims.

Starting from these considerations, the aim of the current study is to analyze the proportion of victimization experiences in children and adolescents aged 6–18 referred to the Meyer Pediatric Hospital in Florence. Specifically, the retrospective study aimed to identify the presence of victimization experience in the records of the children and adolescents' accesses from October 2015 to December 2017 to three services of the Meyer Pediatric Hospital: Functional Pain Clinic (Psychological Department [PD]), Child and Adolescent Psychiatry and Neurorehabilitation (CAPN), Child and Adolescent Abuse Group Unit (GAIA). Differences for ages (6–10 and 11–18 years old) and for type of victimization (physical, verbal, psychological and cybervictimization) will be taken into account.

Specifically, the PD deals with specialized assistance to children, adolescents and their families, in ordinary hospitalization, day hospital and outpatient conditions who are facing a chronic or acute illness, and somatic disorders (e.g., recurrent abdominal pain, dyspepsia, encopresis). The CAPN department deals with diagnosis, care and rehabilitation of children and adolescents with pathologies of psychiatric interest (e.g., depressive disorder, anxiety disorder, eating behavior disorders, posttraumatic stress disorder, somatic disorder). Finally, the GAIA department is a service dedicated to the protection of the rights of minors, in terms of both prevention and detection of suspected cases of abuse, violence and/or maltreatment.

## 2 MATERIAL AND METHODS

### 2.1 Participants

The clinical sample included 705 children and adolescents, 319 males (45.2%) and 386 females (54.8%) accessed at Meyer Pediatric Hospital from October 2015 to December 2017. The age range was from 6 to 18 years old (*M* = 13.09; *SD* = 3.048). 145 subjects (20.6%) belonged to the age category 6–10 years and 560 (79.4%) to the age category 11‐18 years. As regard the nationality, 83.7% of the sample was Italian and 16% was foreign. Sociodemographic and psychopathological data were coded from medical records. Peer victimization information were routinely assessed through medical interview performed on all subjects and their parents, without using instruments specifically aimed to measure these experiences. The experience of victimization in the past medical history was considered occasional when it was mentioned as once during the lifetime present in the clinical record and systematical when it was repeated during the lifetime.

### 2.2 Procedure and analysis of data

The study variables included age, hospital department/unit, type of victimization and experience of victimization. The variables were extrapolated directly from the clinical records, entered in a computerized evaluation matrix, and later imported in the SPSS‐PSW 18 software for statistical analysis. For the prevalence of victimization, we used the frequencies and percentages. To evaluate the impact of victimization within the different Departments of Meyer Pediatric Hospital we used contingency tables and the Chi squared test (*χ* ^2^). Where raw data were lower than expected, Fisher's exact test was used.

## 3 RESULTS

From the analysis of the medical records of the ED, it emerged that among the young people who get access, 108 children and adolescents representing 15.3% of the sample reported to be victimized during the past (PD: 14%; CAPN: 15%; GAIA: 17%), and the remaining 84.7% (*N* = 597) did not. In particular, the victims of occasional bullying are 58 representing 8.2% (26 males and 32 females) of the whole sample and the victims who experienced systematic bullying are 50 representing 7.1% (25 males and 25 females).

Table 1 shows the percentages of victimization in the different Hospital Departments. Among subjects that had access to the PD, 103 (85.1%) were not victimized, 6 (5%) were occasionally victimized and 12 (9.9%) were systematically victimized. Within the CAPN department, 347 (85%) were not victimized, 38 (8.6%) were occasionally victimized and 28 (6.4%) were systematically victimized. Finally, within the GAIA department, 120 (83.3%) were not victimized, 14 (9.7%) were occasionally victimized and 10 (6.9%) were systematically victimized. No significant differences were found between Hospital Departments in relation to no victimization, occasional victimization and systematic victimization (*═* ^2^ = 3.79; *p* = 0.44).

**Table 1:** Victimization within the different Departments of Meyer Pediatric Hospital

**Hospital department**	**No victimization, *N* (%)**	**Occasional victimization, *N* (%)**	**Systematic victimization, *N* (%)**	**Total**	**Sig**.
PD	103 (85.1%)	6 (5%)	12 (9.9%)	121 (100%)	*═* ^2^ (4) = 3.79; *p* = 0.44
CAPN	374 (85%)	38 (8.6%)	28 (6.4%)	440 (100%)	
GAIA	120 (83.3%)	14 (9.7%)	10 (6.9%)	144 (100%)	
Total	597 (84.7%)	58 (8.2%)	50 (7.1%)	705 (100%)	

Abbreviations: PD, Psychological Department); CAPN, Child and Adolescent Psychiatry and Neurorehabilitation; GAIA, Child and Adolescent Abuse Group Unit; Sig., significance.

The effect of age has been tested within each Department separately (see Table 2).

**Table 2:** Victimization within the different age group

**Hospital department**		No victimization, *N* (%)	Occasional victimization, *N* (%)	Systematic Victimization, *N* (%)	Total	Sig.
PD	Age: 6–10	25 (24.3%)	1 (16.7%)	3 (25%)	29	Fisher's exact *p* = 1
	Age: 11–18	78 (75.7%)	5 (83.3%)	9 (75%)	92	
Total		103	6	12	121	
CAPN	Age: 6–10	68 (18.2%)	2 (5.3%)	0	70	Fisher's exact *p* = 0.003
	Age: 11–18	306 (81.8%)	36 (94.7%)	28 (100%)	370	
Total		374	38	28	440	
GAIA	Age: 6–10	38 (31.7%)	6 (42.9%)	2 (20%)	46	Fisher's exact *p* = 0.52
	Age: 11–18	82 (68.3%)	8 (57.1%)	8 (80%)	98	
Total		120	14	10	144	

Abbreviations: PD, Psychological Department); CAPN, Child and Adolescent Psychiatry and Neurorehabilitation; GAIA, Child and Adolescent Abuse Group Unit; Sig., significance.

In relation to the PD, any significant relation was found between the being victimized and the two age groups (Fisher's *p* = 1). The same results have been found for GAIA department (Fisher's *p* = 0.52).

As concern the CAPN department, there was a statistically significant difference between no victimization, occasional victimization and systematic victimization comparing the age group 6‐10 years with the age group 11–18 years (Fisher's *p* = 0.003). Having an age between 11 and 18 is hence linked to a greater possibility of being victims. In particular, 2 (5.3%) subjects for the age group 6‐10 years and 36 (94.7%) for the age group 11–18 years, were occasionally victimized. Instead, 28 (100%) subjects for the age group 11–18 years were systematically victimized.

In relation to the type of victimization, as we can see from Table 3, no significant differences were found between psychological/relational victimization and hospital department (Fisher's *p* = 0.17) and cybervictimization and hospital department (Fisher's *p* = 1). Besides, a statistically significant difference was found between verbal victimization and hospital department (Fisher's *p* = 0.02) and physical victimization and hospital department (Fisher's *p* < 0.001). Having access to the CAPN department is hence linked to a greater possibility to be verbally victimized, specifically, 35 (63.6%) subjects of CAPN department, 11 (20%) of GAIA department, and 9 (16.4%) of PD. Moreover, having access to GAIA department is hence linked to a greater possibility to be physically victimized, specifically, 20 (54.1%) subjects of GAIA department, 12 (32.4%) of CAPN department, and 5 (13.5%) of PD.

**Table 3:** Type of victimization within the different Departments of Meyer Pediatric Hospital

**Type of victimization**	**Verbal, *N* (%)**	**Psychological and relational, *N* (%)**	**Physical, *N* (%)**	**Cyber, *N* (%)**	**Total**	
PD	9 (16.4%)	4 (16%)	5 (13.5%)	0		18
CAPN	35 (63.6%)	20 (80%)	12 (32.4%)	3 (100%)		70
GAIA	11 (20%)	1 (4%)	20 (54.1%)	0		32
Total	55	25	37	3		120
Sig.	Fisher's exact *p* = 0.02	Fisher's exact *p* = 0.17	Fisher's exact *p* < 0.001	Fisher's exact *p* = 1	

Abbreviations: PD, Psychological Department); CAPN, Child and Adolescent Psychiatry and Neurorehabilitation; GAIA, Child and Adolescent Abuse Group Unit; Sig., significance.

## 4 DISCUSSION

Overall, 15.3% of the subjects from 6 to 18 years old who accessed to the Meyer Pediatric Hospital in Florence from October 2015 to December 2017 reported victimization episodes. Specifically, 8.2% reported occasional victimization and 7.1% systematic victimization. This finding differs from the data in the literature, showing lower values of peer victimization in the Italian sample as compared to other studies attesting around 19%–29% (Alavi et al., 2015; Hansen et al., 2014; Luukkonen et al., 2010; Roberts et al., 2016; Salmon et al., 2000). Different possible explanations can be advanced. First, the time period considered for the analyses. The access period to the EDs reported by the literature is heterogeneous, ranging from 2001 to 2015. Besides, the number of years examined ranged from 2 years (Roberts et al., 2016), 3 years (Hansen et al., 2014), 4 years (Alavi et al., 2015), to 5 years (Luukkonen et al., 2010). A second important explanation includes the specific ED considered in the studies. The majority of the studies considered the child and adolescent psychiatry department, whereas in our case we have three different EDs, characterized by different health needs. The age of the patients presented also a relevant heterogeneity. In the majority of the studies (Hansen et al., 2014; Luukkonen et al., 2010), adolescents, and not children, were considered. This contributes to register lower prevalence rates.

Regarding the presence of victimization into the three services of the Meyer Pediatric Hospital any significant difference was found. In fact, we can see how the percentage of victims in the different departments is roughly the same.

Concerning the age group, we found that adolescents aged between 11 and 18 years accessing to CAPN department, are more likely to be victims as compared to children aged between 6 and 10. This result confirms that adolescents are the main targets for peer victimization (Nocentini et al., 2013; Pellegrini & Long, 2002). Interesting, no significant difference was found for the PD and GAIA department. Overall, these findings stressed a specificity trend for consequences of victimization in relation to the age. Psychosomatic symptoms and physical injuries are typical for both children and adolescents, whereas psychiatric symptoms are more frequent in adolescents' Italian victims.

The type of victimization seems to be associated with the access department. In particular, having access to the CAPN department is associated to a greater possibility to be verbally victimized. This could be explained by the fact that those who are verbally victimized are more prone to have psychopathological symptoms treated predominantly from the CAPN. According to the literature, specific mental health and psychopathological conditions (i.e., intellectual disability, learning disabilities, eating disorders, anxiety symptoms) are associated with a higher probability to be victimized, although the longitudinal association is likely to be bidirectional (Catone et al., 2017; Kwan et al., 2017; Maiano et al., 2016). On the other hand, having access to GAIA department highlights a clear association with physical victimization. This department, in fact, deals with cases of abuse and maltreatment from family, other adults and from peers, and therefore it is more likely that those children and adolescents with physical consequences had an access in this ED.

Overall, the current study contributes to the literature on the victimization in children and adolescents accessing to an ED in three directions. First, the consideration of a larger age span, including not only adolescents but also children from 6 years old. Second, the consideration of different EDs and finally the consideration of different types of victimization. Interrelations between these factors allowed to give more insights about the consequences and the nature of the victimization in children and adolescents referred to an ED.

Implications of these results can be defined in terms of assessment and of tailoring interventions. Findings suggest the importance of an accurate assessment of victimization experiences in the clinical history of children and adolescents with access to an ED of a Pediatric Hospital. Protocols of tailored interventions can be activated for the short term and the long term, together with the community mental health services and schools. This procedure can prevent future re‐victimization and crystallization of symptoms across time.

### 4.1 Limitations and future studies

The most relevant limitation of the study is the lack of a systematic evaluation of the experiences of peer victimization that have been extrapolated from medical interview performed on all subjects and their parents. Future studies should assess peer victimization experiences with appropriate and specific scales. Nevertheless, this study represents one of the first Italian study about peer victimization amongst children and adolescents referred by Pediatric ED for physical injuries, psychological and psychosomatic symptoms (Mental Health Emergency). The second limitation consists of the lack of the prospective design. Excepting for the GAIA. department treating physical injuries, our data did not allow to establish a causal relationship between victimization and access to the hospital or the onset of the psychopathology. Future studies might profit by a more accurate assessment of victimization experience for each child and adolescent who have access to the ED using specific instruments and procedures.

## CONFLICT OF INTERESTS

The authors declare that there are no conflict of interests.

## ETHICS STATEMENT

The study was approved by the Pediatric Ethics Committee of the Tuscany Region (Parental and child informed consent were obtained). Data were collected between January 2016 through December 2017. A. Meyer Children's Hospital, Florence and University of Florence, Italy. Pediatric Ethics Committee of the Tuscany Region provided ethical approval for the study (CEP 168/2017).

## Data Availability

Data are not available according to the statement approved by the Ethical Committee.
